# Preparation and Evaluation of Antioxidant Activities of Bioactive Peptides Obtained from *Cornus officinalis*

**DOI:** 10.3390/molecules27041232

**Published:** 2022-02-11

**Authors:** Xin Zhang, Hao Zhang, Pengfei Jiao, Mengrong Xia, Bo Tang

**Affiliations:** 1 School of Life Science and Agricultural Engineering, Nanyang Normal University, Nanyang 473061, China; zhanghao660@nynu.edu.cn (H.Z.); jiaopf@nynu.edu.cn (P.J.); xmr13271395521@163.com (M.X.); 2 Research Center of Henan Provincial Agricultural Biomass Resource Engineering and Technology, Nanyang 473061, China; 3 College of Food and Bioengineering, Bengbu University, Bengbu 233030, China; 3120170650@bit.edu.cn

**Keywords:** peptides, response surface methodology, antioxidant, *Cornus officinalis*

## Abstract

The present study is a preparation of bioactive peptides from *Cornus officinalis* proteins by the compound enzymatic hydrolysis method. Response surface methodology (RSM) coupled with Box–Behnken design (BBD) is used to optimize the preparation process of *Cornus officinalis* peptides. The effects of independent variables, such as the amount of enzyme, pH value, time, extraction times and the ratio of material to liquid on the yield of peptides, are also investigated. The analysis results of the RSM model show that the optimum conditions for the extraction of *Cornus officinalis* peptides were a pH value of 6.76, temperature of 48.84 °C and the amount of enzyme of 0.19%. Under optimal conditions, the yield of peptides was 36.18 ± 0.26 %, which was close to the predicted yield by the RSM model. Additionally, the prepared *Cornus officinalis* peptides showed significant antioxidant activity; the scavenging rates of the peptides for DPPH and ·OH were 48.47% and 29.41%, respectively. The results of the cell proliferation assay revealed that the prepared *Cornus officinalis* peptides could promote embryo fibroblast cells proliferation and repair oxidative damage cells. These results have a practical application value in the design of novel functional food formulations by using *Cornus officinalis*.

## 1. Introduction

*Cornus officinalis* (*Cornus officinalis Sieb.et Zucc.*) is classified as a tonic in traditional Chinese medicine. There are various biologic active compounds, including iridoids, tannins, flavonoids, polysaccharides, triterpenoids, ursolic acid and organic acid esters, are present in *Cornus officinalis* [1,2,3]. Pharmacological studies have revealed that the *Cornus officinalis* possesses the effects of being anti-neoplastic, hypoglycemic, enhancing sperm motility, antioxidation and being anti-inflammatory [4,5,6,7,8]. At present, there are many studies on the extraction of polysaccharides, phenolic acid compounds, diverse iridoid glycosides, saponins, tannins and other physiological active substances from *Cornus officinalis* [9,10,11,12]. However, there are only a few studies on how to use the *Cornus officinalis* protein [13,14,15]. The protein resources in *Cornus officinalis* are often wasted during the extraction process. In fact, proteins from *Cornus officinalis* have a high nutritional value and contain a variety of essential amino acids. Therefore, it is significant to develop an effective strategy to utilize protein resources from *Cornus officinalis*.

Peptides hydrolyzed from proteins have diverse biological activities, including antioxidant, cholesterol-lowering, blood pressure-lowering, and antibacterial capacities [16,17,18]. Compared with proteins, peptides facilitate digestion and absorption by the human body [19]. The protein in *Cornus officinalis* is coated with cellulose and has antigenicity, low solubility and high viscosity, which are adverse to human absorption and digestion. Therefore, the development of a bioactive peptide is an effective approach to make use of protein resources from *Cornus officinalis*. The peptide from *Cornus officinalis* has diversity in the structural properties that possess potential commercial value to be developed into novel functional food products. The preparation methods of plant bioactive peptides include chemical methods, enzymatic methods and microbial fermentation methods, etc. [20,21,22]. The enzymatic hydrolysis method is mild and easy to control, and most of the hydrolytic products are short peptides and amino acids, which conform to the requirements of food hygiene [23,24]. Therefore, bioactive peptide prepared by enzymatic hydrolysis is an effective way to transform proteins for its diversification for function foods. For the preparation of *Cornus officinalis* peptides, the enzymatic hydrolysis conditions are particularly important. The appropriate conditions of enzymatic hydrolysis directly affect the size, quantity and amino acid composition of the peptide in the product, and then affect the biological activity and functional properties of the hydrolysate [25]. The key to produce functional peptides is to control the hydrolysis conditions, such as pH, temperature, ratio of enzyme to protein, hydrolysis time, etc. [26,27].

Based on this, the present study develops a technology to prepare *Cornus officinalis* bioactive peptides. *Cornus officinalis* proteins were isolated by a solubilization precipitation method and optimized to improve the yield of *Cornus officinalis* protein. The obtained proteins were hydrolyzed by multiple enzymes to prepare bioactive peptides. Single-factor method and response surface methodology were carried out to identify interactions among various variables in the preparing process [28,29]. The process conditions are analyzed and optimized by the designed mathematical model and equation. Moreover, the clearance rates of 1,1-diphenyl-2-trinitrophenylhydrazine (DPPH) and ·OH were determined to evaluate the antioxidant of the prepared *Cornus officinalis* peptides [30,31]. The effect of *Cornus officinalis* peptides on cell proliferation in vitro was also studied using the ethyl thiazolyl tetrazolium (MTT) method. These prepared peptides not only inherit the function and activity of *Cornus officinalis* proteins, but also have the advantages of good solubility, having antioxidant properties, facilitating digestion and being rich in essential amino acids. The results of the study provide value information for the development of functional products in food, cosmetic, health care and medicinal fields using *Cornus officinalis*.

## 2. Materials and Methods

### 2.1. Material and Reagents

Cornus officinalis was obtained from the local market (Nanyang, China). Sodium chloride (NaCl), sodium hydroxide (NaOH) and hydrogen peroxide (H_2_O_2_) were purchased from Tianjin Fengchuan Chemical Reagent Co. Ltd. (Tianjin, China). Papain and trypsin were purchased from Hefei Bomei Biotechnology Co. Ltd. (Hefei, China). 1,1-Diphenyl-2-trinitrophenylhydrazine (DPPH), fetal bovine serum (FBS), tri-nitro-benzene-sulfonic acid (TNBS), dimethyl sulfoxide (DMSO), ferric chloride, tripyridyl-s-triazine (TPTZ) and flavourzyme were received from Macklin Biochemical Co. Ltd. (Shanghai, China). High glucose medium (DMEM) and 3-(4,5-Dimethyl-2-thiazolyl)-2,5-diphenyl-2H-tetrazolium bromide (MTT) were purchased from Sigma Aldrich Co. Ltd. (Shanghai, China). A chicken embryo fibroblast (CEF) was obtained from Longyue Biotechnology Co. Ltd. (Beijing, China). UV–Vis spectrophotometer (Analytikjena Specord 210 plus, Jena, Germany). High performance liquid chromatography (HPLC, Shimadzu LC20AT, Tokyo, Japan). Liquid chromatograpy mass spectrometry (LC-Mass, Thermo TSQ, Waltham, MA, USA). 

### 2.2. Extraction of Protein from Cornus officinalis

*Cornus officinalis* protein was extracted by alkali extraction and acid precipitation method [32,33]. In brief, the *Cornus officinalis* was dried and smashed to produce *Cornus officinalis* powder. A total of 10 g of the powder was dispersed in 1.0% NaCl solution and ultrasonicated for 30 min. Then, the pH value of the solution was adjusted to 9.0. After the reaction at 35 °C for 3 h, the supernatant was collected by centrifugation and the pH value was adjusted to 4.5 overnight in refrigerator. The *Cornus officinalis* protein was collected by centrifugation and vacuum dried.

### 2.3. Preparation of Cornus officinalis Peptides

The protein of *Cornus officinalis* was hydrolyzed by double enzyme to prepare peptides. In brief, 5.0 g of *Cornus officinalis* protein powder was dispersed in water and different mass of papain and trypsin were added. The supernatant was collected by centrifugation after hydrolysis, and the peptide powder of *Cornus officinalis* was prepared by vacuum freeze drying. The molecular weight of the obtained peptides was determined by HPLC method using TSK-GEL G2000 SW (300 × 7.8 mm, 5 μm, 125 Å). After drying process, the yield (%) of peptides was calculated using the following Equation (1):

(1) $$\mathrm{Yield}\;\mathrm{of}\;\mathrm{peptides}\;(\%)=\frac{\mathrm{Weight}\;\mathrm{of}\;\mathrm{peptides}}{\mathrm{Weight}\;\mathrm{of}\;\mathrm{Cornus}\;\mathrm{officinalis}\;\mathrm{protein}}\times 100$$

#### 2.3.1. Single-Factor Experiments

The effects of the amount of papain (A), trypsin (B), flavor protease (C) and three complex enzymes (A:B = 1:1; A:C = 1:1; B:C = 1:1, *w*/*w*) on the degree of protein hydrolysis in *Cornus officinalis* were investigated. The other reaction conditions were fixed at the solid–liquid ratio of 1:20, reaction time of 3.5 h, reaction temperature 50 °C, and initial pH value of 6.0. The hydrolysis efficiency of *Cornus officinalis* proteins were evaluated by degree of hydrolysis (DH). The DH was measured by the TNBS method [34], and calculated as follows using Equation (2):

(2) $$\mathrm{DH}\;(\%)=\frac{L_{t}-L_{0}}{L_{max}-L_{0}}\times 100$$

where *L_t_* is the content of specific liberated amino acid at time *t*; *L*_0_ is the initial content of the specific amino acid; and *L_max_* is the maximum content of the specific amino acid after hydrolysis by enzyme.

A series of single-factor experiments were carried out to investigate the effect of the different factors on the yield of *Cornus officinalis* peptides. The effects of parameters including the substrate ratio of enzymes (0.05~1.0%, *w*/*v*), solid-to-liquid ratio (1:5~1:30, *w*/*v*), pH value of the solution (4.0~10.0), enzymatic hydrolysis temperature (25~80 °C), and hydrolysis time (0.5~6 h) on the yield of *Cornus officinalis* peptides were investigated. 

#### 2.3.2. RSM Design for Optimization of the Yield of Peptides

A three-level, three-factor RSM was carried out to optimize the process to obtain the maximum yield of peptides from *Cornus officinalis*. The parameters of the amount of enzyme (mass ratio, %), pH value and reaction temperature (°C) were selected according to the results of single-factor experiment. The yield (%) of peptides was chosen as the response of the experiment. Table 1 showed that the level of each factor and range of independent variables. Table 2 shows the results of the designed experiments with independent variables at variant levels.

### 2.4. Determinde of Antioxidant Activity

#### 2.4.1. DPPH Radical Scavenging Activity

The DPPH radical scavenging activity was determined according to previously reported methods [35] to examine the antioxidant activity of *Cornus officinalis* peptides. Briefly, the prepared *Cornus officinalis* peptides were dissolved in deionized water. A series of concentrations (0.1~1.0 mg/mL) of the sample solution were mixed with 2.5 mL DPPH. After being incubated for 30 min, the absorbance at 570 nm of the resulting solution was measured (*A_sample_*) and methanol was used as control (*A_control_*). The DPPH radical scavenging capacity of the *Cornus officinalis* peptides was calculated by the following Equation (3):

(3) $$\mathrm{DPPH}\;\mathrm{scavenging}\;\mathrm{capacity}\;(\%)=\frac{A_{control}-A_{sample}}{A_{control}}\times 100$$

#### 2.4.2. Hydroxyl Radical (·OH) Scavenging Activity

The scavenging ability to hydroxyl radical of *Cornus officinalis* peptides was determined according to reported method with some modification [36]. In brief, a ferrous sulfate solution (5 mL, 1 mmol/L) was mixed with 8 mL salicylic acid-ethanol solution (3 mmol/L), and then a hydrogen peroxide solution (5 mL, 3 mmol/L) was added. A total of 2 mL of *Cornus officinalis* peptides solution at different concentrations (0.1~1.0 mg/mL) was mixed. After being incubated at 37 °C for 1 h, the absorbance at 510 nm of the supernatant was determined (*A*_1_). Deionized water was used as control (*A*_0_). The ·OH radical scavenging capacity of the *Cornus officinalis* peptides was calculated using the following Equation (4):

(4) $$\mathrm{OH}\;\mathrm{scavenging}\;\mathrm{activity}\;(\%)=\left(A_{0}-A_{1}\right)/A_{0}\times 100$$

#### 2.4.3. Ferric Reducing Antioxidant Power (FRAP) Assay

The antioxidant power of the prepared peptides was measured by the FRAP assay [37]. A total of 2.5 mL FeCl_3_·6H_2_O (20 mmol/L), 2.5 mL TPTZ (10 mmol/L) and 25 mL acetate buffer (300 mM, pH 3.6) were mixed and incubated at 37 °C. The absorbance at 593 nm of the Fe^3+^/TPTZ complex was determined (*A*_0_). Then, the different concentrations of peptides solution were mixed. After being incubated for 8 min, the absorbance of the solution at 593 nm was determined (*A*_1_). The FRAP value was calculated by the increase in the absorbance of Fe^3+^/TPTZ complex (Δ*A*, Δ*A* = *A*_1_ − *A*_0_). 

### 2.5. Cell Proliferation Experiments

The effects of different concentrations of *Cornus officinalis* peptides on CEF cell proliferation were tested by MTT method [38]. Briefly, CEF cells in the logarithmic phase were inoculated into 96-well plates at 8 × 10*^−^*^3^ density and 100 μL of different concentrations of *Cornus officinalis* peptides were added. After incubation for 24 h and elution with phosphate buffer (pH 7.4), 20 μL MTT solution (5 mg/mL) was added. It was incubated for another 4 h, and 100 μL DMSO was added to stop the reaction. An MTT solution was used as blank. The optical density at 490 nm of the sample was measured (*OD_sample_*). The control group was created using the same procedure, but with added 100 μL DEME (*OD_control_*). The cell viability (%) was calculated by the following Equation (5):

(5) Cell viability (%)=ODsampleODcontrol×100

### 2.6. Effect of Peptides on Oxidized Damaged CEF Cell

The ability of *Cornus officinalis* peptides to protect human cell against oxidative damage was evaluated. The oxidative damage model of CEF cells was established by hydrogen peroxide (H_2_O_2_) induced in vitro [39]. CEF cells in the logarithmic phase were inoculated in 96-well plates at 8 × 10*^−^*^3^ density. A total fo 100 μL of different H_2_O_2_ solution was added to the well and incubated for 1.5 h. Each well was washed with phosphate buffer (pH 7.4) and then 100 μL DEME solution was added. After incubation for another 24 h, the cell viability was calculated. No H_2_O_2_ of the solution added was used as blank. The experiments used 70% of the blank viability as oxidative damage model of CEF cells. A total of 100 μL of different concentration (0.05~0.8 mg/mL) of the peptide solution was added into the oxidized damaged CEF cells. The DEME solution was used as control, and the cell viability was measured after incubation for 24 h.

## 3. Results

### 3.1. Optimization the Conditions of Protein Isolated from Cornus officinalis

**Figure 1:**
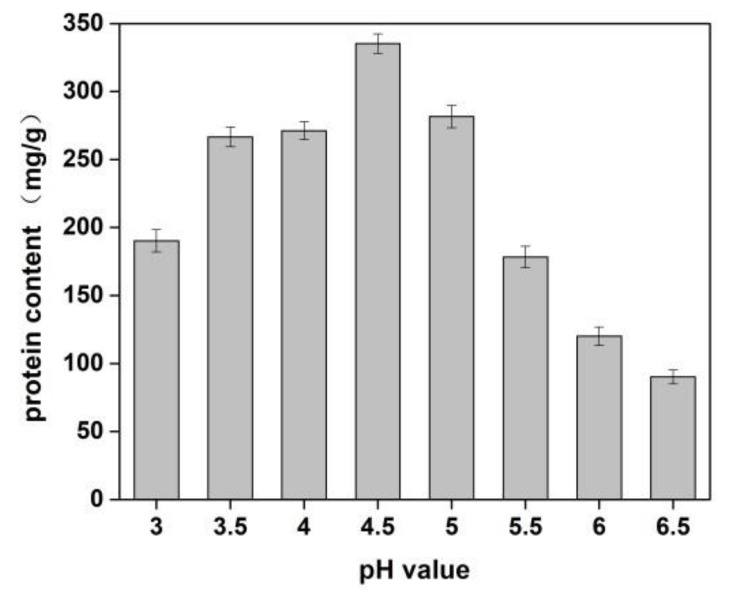
Effects of pH value on the yield of proteins of *Cornus officinalis.* Experimental conditions: 10 g *Cornus officinalis* powder in 1.0% NaCl solution, at 35 °C.

The effects of sodium chloride concentration and pH value of solution on the yield of protein from *Cornus officinalis* were investigated. As shown in Figure 1, a higher protein yield was achieved at the pH value was 4.5. It is because of the pH value of the solution was close to the isoelectric point of the protein, which resulted in protein precipitation. The better protein yield was obtained when the concentration of NaCl was 1.0%. It is seen that a lower concentration of neutral salt can effectively increase the surface charge of proteins and enhance the interaction with water molecules, which improves the solubility of proteins [40]. Moreover, Na^+^ ions in dilute salt solution can remove impurities in the *Cornus officinalis* solution and partially combine with proteins to prevent protein denaturation.

### 3.2. Single-Factor Experiments Effects of Enzyme on the Yield of Cornus officinalis Peptides

**Figure 2:**
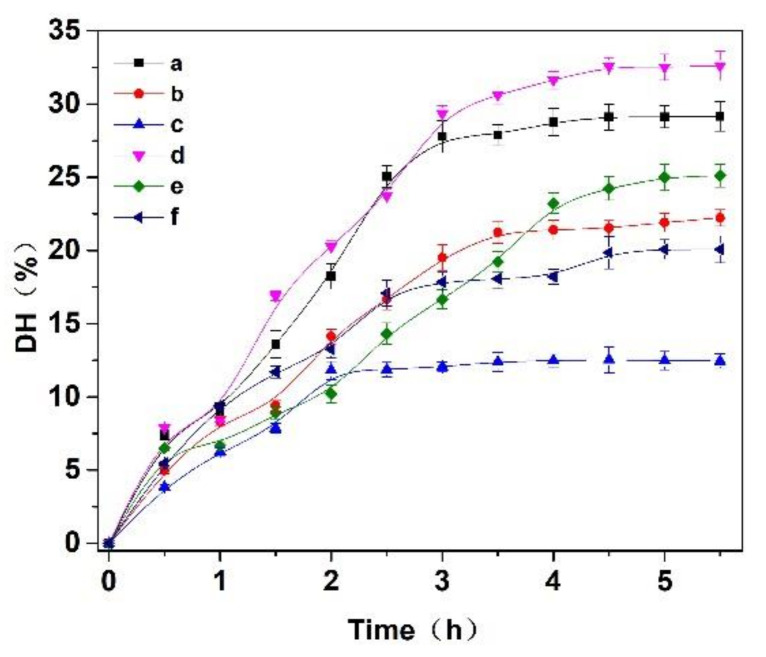
Effects of (a) papain, (b) trypsin, (c) flavourzyme, (d) papain:trypsin (1:1, *w*/*w*), (e) papain:flavourzyme (1:1, *w*/*w*) and (f) trypsin:flavourzyme (1:1, *w*/*w*) on the hydrolysis degree of the *Cornus officinalis* protein. Experimental conditions: the solid–liquid ratio of 1:20, reaction temperature 50 °C, and initial pH value of 6.0.

Three enzymes, trypsin, flavourzyme and papain, were selected to hydrolyze the *Cornus officinalis* protein. As shown in Figure 2, the hydrolysis degree of the *Cornus officinalis* protein by papain (A), trypsin (B), flavor proteinase (C) and three complex enzymes (A:B = 1:1; A:C = 1:1; B:C = 1:1, *w*/*w*) increased with the extension of time. The DH of the protein becomes stable at about 3.5 h, indicating that the proteolysis of the *Cornus officinalis* protein was complete. The results showed that the combination of papain and trypsin enzymes (A:B) had the best hydrolysis effect on *Cornus officinalis* protein with the DH was 32.62%. Therefore, papain and trypsin were combined to hydrolyze *Cornus officinalis* protein for prepare peptides. Additionally, the effect of the ratio between papain and trypsin on the hydrolysis of the *Cornus officinalis* protein was also tested, and the analytical result indicates that the maximum of DH of the *Cornus officinalis* protein occurs when the ratio of the papain and trypsin was 6:4.

**Figure 3:**
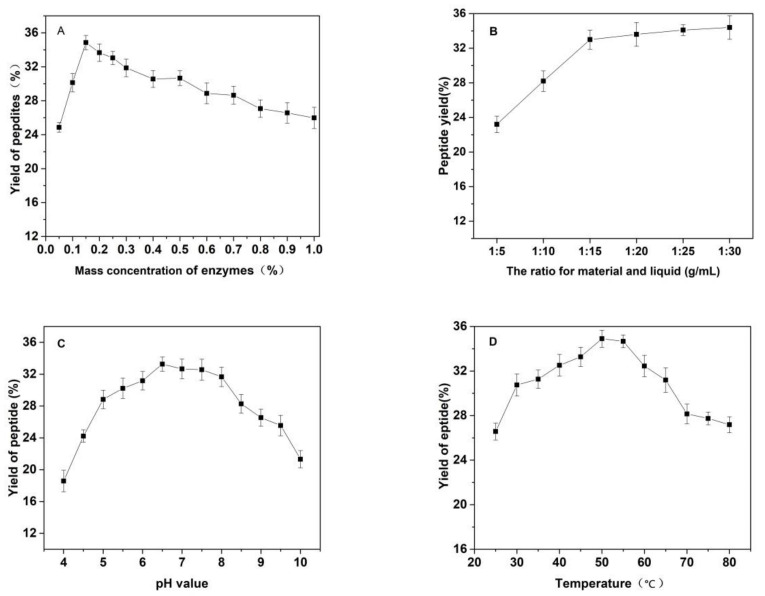

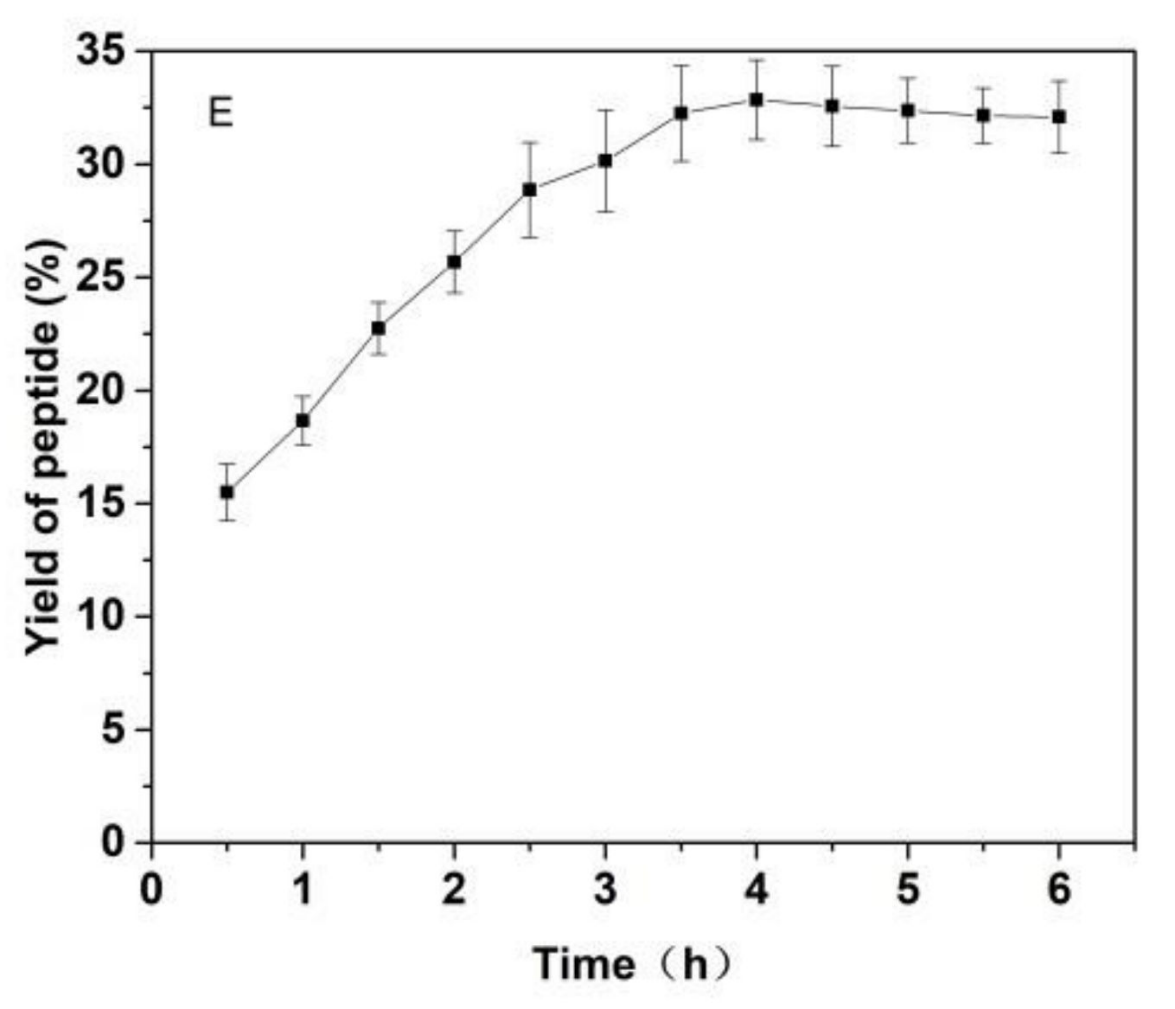
Effects of (**A**) the substrate ratio of enzymes (%), (**B**) the ratio for materials and liquid (g/mL), (**C**) pH, (**D**) temperature (°C) and (**E**) reaction time (h) on the yield of *Cornus officinalis* peptides.

As shown in Figure 3, the effects of the substrate ratio of enzymes, the ratio for materials and liquid, pH value of solutions, temperature and reaction time on the yield of *Cornus officinalis* peptides were investigated. The yield of *Cornus officinalis* peptides increased significantly when the mass concentration of enzymes was in the range of 0.05–0.20%. The highest yield of peptides was achieved when the mass concentration of enzyme was 0.15% (Figure 3A). However, the yield of peptides gradually decreases when the concentration of enzyme concentration continues to be raised. This may be because of an excessive amount of enzymes inhibits the transformation of intermediate products to end products, resulting in a decline of enzymatic hydrolysis efficiency. As shown in Figure 3B, the yield of peptides gradually increased with the decrease in the solid–liquid ratio. The yield of peptides becomes stabilized when the solid–liquid ratio reaches 1:20. Due to the larger solvent volume not being conducive to concentration and drying in the actual operation and energy consumption, the solid–liquid ratio of 1:15 was selected for the preparation of *Cornus officinalis* peptides. The initial pH value of the solution is very important for the enzymatic hydrolysis reaction. In the process of the hydrolysis of *Cornus officinalis* protein, the pH value directly affects the dissociation state of the substrate and the group dissociation of the active part of the enzyme. The optimum pH for papain is in the range of 5.0 to 7.0 and for trypsin is in the range of 7.6 to 9.0; however, the substrate degrades easily under alkaline conditions and a lower pH is favorable for proteolytic peptides [41]. The initial pH value of the enzymatic hydrolysis was studied. As shown in Figure 3C, the peptide yield is higher when the pH value of the solution is in the range of 5.5–8.0, and the highest peptides yield reached at the pH value of the solution is 6.5. Therefore, the initial pH of solution was set to be 6.5 for the hydrolysis of *Cornus officinalis* protein. As shown in Figure 3D, the yield of *Cornus officinalis* peptides increased gradually with the increase in temperature in the range of 25–50 °C. When the temperature continued to rise, the yield of peptides decreased and gradually stabilized at 70 °C. This is because the catalytic efficiency of enzyme is accelerated in higher temperatures, which increases the yield of peptides. However, although papain has strong heat resistance, trypsin is easy to self-hydrolyze at high temperatures, resulting in the loss of enzymatic activity and affecting the efficiency of enzymatic hydrolysis. These results verified that the optimal reaction temperature for the enzymatic hydrolysis of *Cornus officinalis* proteins was 50 °C. The yield of peptides increased quickly with the increasing reaction time. After reaction for 3.5 h, the peptides yield reached the highest, and then the peptides yield became stable. Therefore, the optimal reaction time for preparation of *Cornus officinalis* peptides was 3.5 h.

### 3.3. Optimization of the Preparation of Cornus officinalis Peptides by RSM Method 

In this study, the prediction model was established by the RSM method and the conditions of preparation of peptides from *Cornus officinalis* were optimized. The independent variables selected for the optimization of the preparation process included the pH value, temperature and the amount of enzymes. Table 2 provides the RSM design matrix and the code values of run experiments with corresponding results. The results were used to evaluate the effects of the three independent variables on the yield of peptides. Table 3 displays a sequential model summary for the yield of peptides. These results show that the quadratic model was the most fit for yield of peptides, according to the *p* value (*p* < 0.05), lack of fit (*p* > 0.05) and R^2^ value (0.9670) of the model [42]. The mathematical equation describing the yield (%) of peptides was given by following quadratic Equation (6):

Y = 35.28 + 0.4590 A + 0.8120 B × 0.6597 C × 1.10 AB × 0.7250 AC + 0.4800 BC × 2.27 A^2^ × 2.36 B^2^ × 0.6768 C^2^ (6)

where Y is the yield (%) of peptides, while A, B, and C are pH value, temperature (°C) and the amount of enzyme (%), respectively.

**Figure 4:**
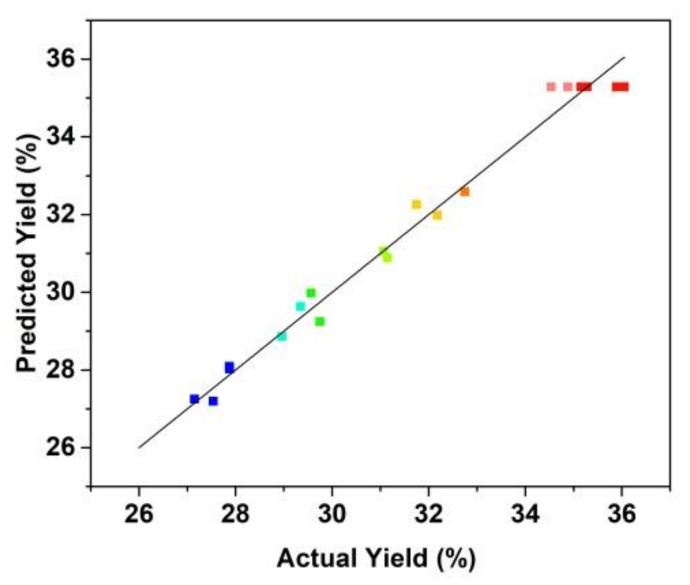
Correlation of actual and predicted yield of the prepared *Cornus officinalis* peptides (%).

Figure 4 shows that the fitting plot for the predicted and actual values of peptide yield. The fitting results show that the regression model was significant with R^2^ value was 0.9670 and the lack of fit value of the model was 0.5627 (>0.05, not significant). The R^2^ value of the regression model indicated that the fitted plot could explain 96.70% of the parameters of the model.

The analysis of variance (ANOVA) of model for the yield of peptides is shown in Table 4. As shown in Table 4, the F-value of the model was 59.54 with a very small *p*-value (*p* < 0.0001), which indicated that the generated model was significant and could describe the relationship between the parameters well [43]. Each value of the significance of the parameters in the model was obtained using F- and p-test. Data obtained showed that two linear terms (B and C) and interaction terms (AB and AC) produced a significant effect (*p* < 0.001) on the yield of peptides. The ANOVA results suggest that the designed RSM model can be used for the preparation of peptides from *Cornus officinalis*.

### 3.4. Response Surface Analysis

**Figure 5:**
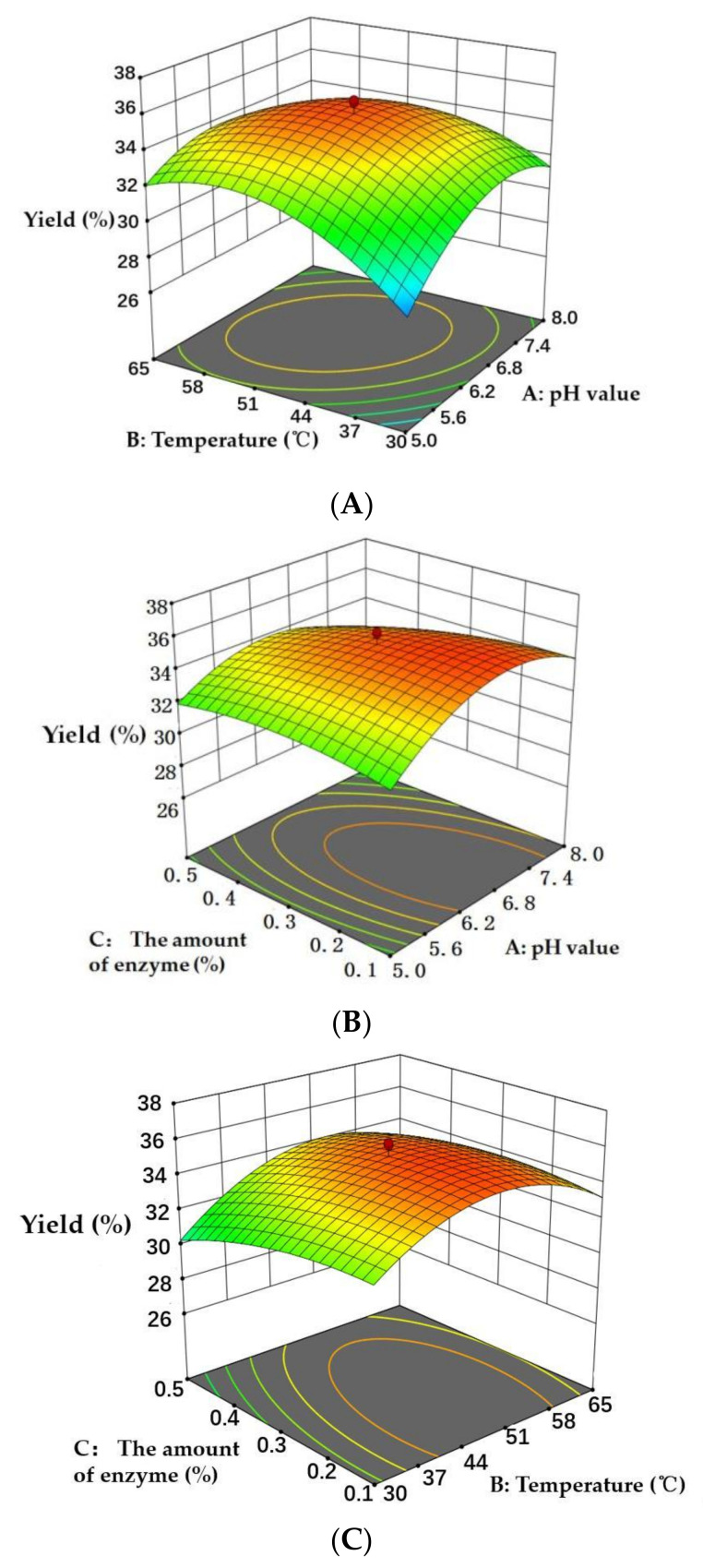
Three-dimensional response surface graphs of (**A**) temperature (°C) and pH value, (**B**) pH value and the amount of enzyme (%), and (**C**) temperature (°C) and the amount of enzyme (%) on the yield of peptides.

The three-dimensional (3D) response surface graphs produced by the designed model (Figure 5) showed the visual effects of the experimental variable levels on the response, relationship and interaction of two independent variables. In the 3D response surface graphs, two of the experimental variables were displayed and another parameter was fixed (0 level). The 3D graphs were used to evaluate the optimal level of variables for the preparation of *Cornus officinalis* peptides. As shown in Figure 5, the interactions of pH value and temperature, the pH value and the amount of enzyme, and the temperature and the amount of enzyme are presented in Figure 5A–C, respectively. The 3D response surface graphs revealed that the temperature and the amount of enzyme showed a higher significant influence on the yield of peptides than that of pH value. Based on the experimental results, the designed RSM model was successful to optimize the extraction process to obtain the maximum yield of peptides from *Cornus officinalis*.

### 3.5. Optimization of Parameters and Verification of the Model

According to the results of response surface analysis, the optimal conditions for the preparation of peptides were the pH value of initial solution of 6.76, the reaction temperature of 48.84 °C, and the amount of enzyme of 0.19%. Under optimal conditions, the maximum yield of peptides predicted by the model was 35.54%. The actual extraction of the yield of peptides was recorded as 36.18 ± 0.26 %, which was highly consistent with the predicted yield. These results showed that the RSM model has high reliably and accurately predicts the relationship between the preparation variables and the yield of peptides. Most of the molecular weight (MW) of *Cornus officinalis* peptides (72.57%) was less than 1000 Da (Table 5). It can be seen that the prepared peptides by the compound enzymatic hydrolysis of *Cornus officinalis* protein are oligopeptides, which possess higher medical and commercial value.

### 3.6. LC-MASS Analysis

**Figure 6:**
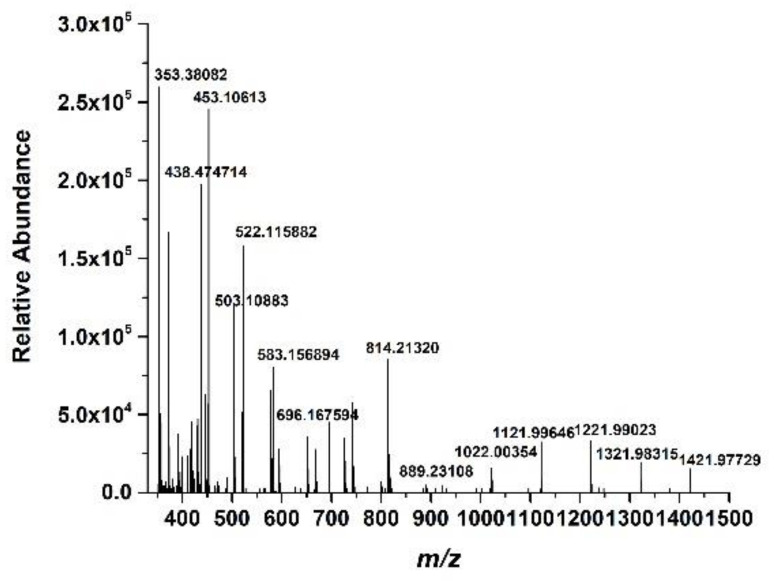
The mass spectrum of the prepared *Cornus officinalis* peptides.

The LC-MASS analysis was carried out to further identify the structure of the prepared *Cornus officinalis* peptides; the results are shown in Figure 6. As shown in Figure 6, for peptide identification, the prepared *Cornus officinalis* peptides were subjected to mass spectrometry. The manual analysis gave possible sequences of fraction: Leu-Ala-Asn (353.3 Da), Ile-Ala-Asn (353.3 Da); Ile-Pro-Pro-Leu (438 Da); Arg-Lys-Arg (458 Da); Phe-Leu-Pro-Phe (522.64 Da); Pro-Gln-Glu-Val-Leu (584.4 Da); Lys-Phe-Ala-Leu-Pro-Gln (702 Da); Val-Leu-Asn-Glu-Asn-Leu-Leu (813 Da); and Leu-Pro-Gln-Asn-Ile-Pro-Pro-Leu (890.5 Da) [44,45,46,47].

### 3.7. Antioxidant Activity of the Prepared Peptides

**Figure 7:**
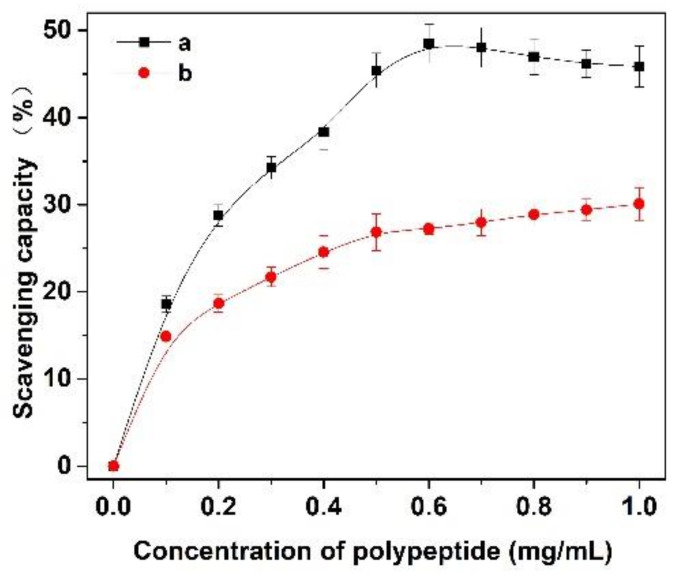
The scavenging capacity (%) of (a) DPPH and (b) ·OH of *Cornus officinalis* peptides.

**Figure 8:**
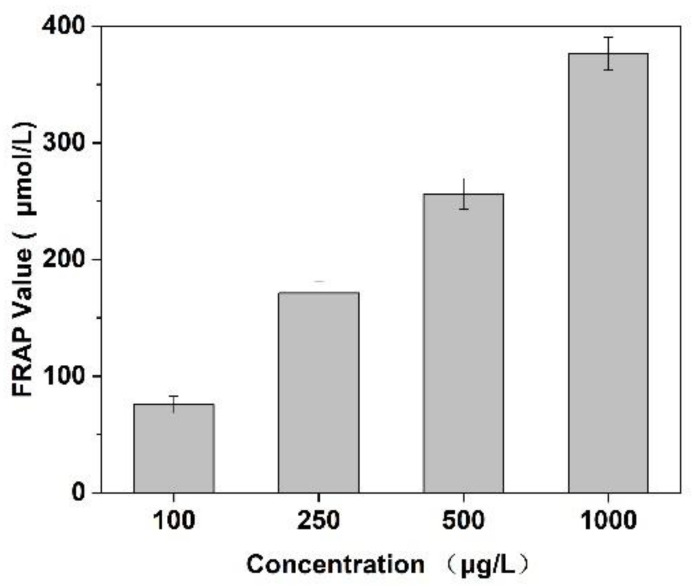
Ferric-reducing antioxidant power assay of *Cornus officinalis* peptides. All values are mean ± SD (*n* = 3).

In this experiment, the scavenging capacity of DPPH and Fe^2+^/H_2_O_2_ in the system was used to evaluate the antioxidant activity of *Cornus officinalis* peptides. As shown in Figure 7, the prepared peptides presented evident antioxidant activity with the increasing concentration of peptides. The clearance rates of peptides for DPPH and ·OH were 48.47% and 29.41%, respectively. The results suggest that the prepared *Cornus officinalis* peptides had good free radical scavenging ability due to the *Cornus officinalis* peptides being able to generate more electrons to react with free radicals [48]. The ferric-reducing ability of the prepared peptides was determined by the FRAP assay. As shown in Figure 8, the concentration of the peptides is proportional to the FRAP values of the antioxidants in the sample. The *Cornus officinalis* peptides showed significant FRAP values, indicating that the prepared *Cornus officinalis* peptides have a good ferric-reducing ability [49].

### 3.8. Effect on CEF Cell Proliferation 

**Figure 9:**
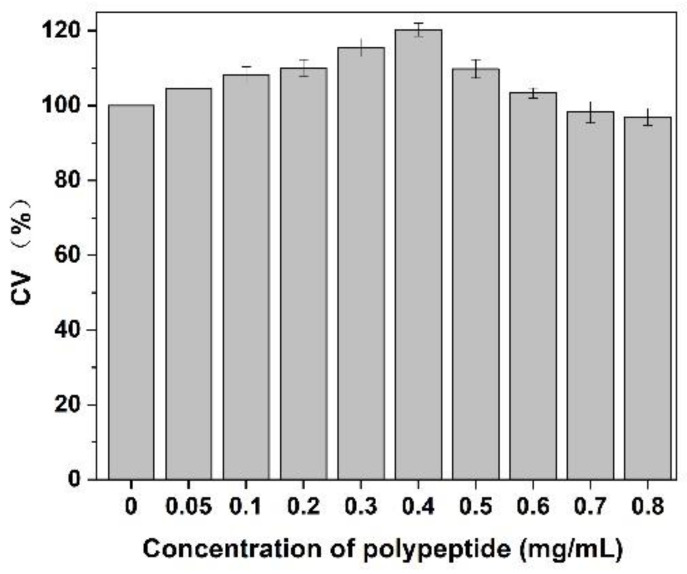
The effect of different concentrations of *Cornus officinalis* peptides on CEF cell proliferation. Experimental conditions: phosphate buffer (pH 7.4), at 37 °C.

**Figure 10:**
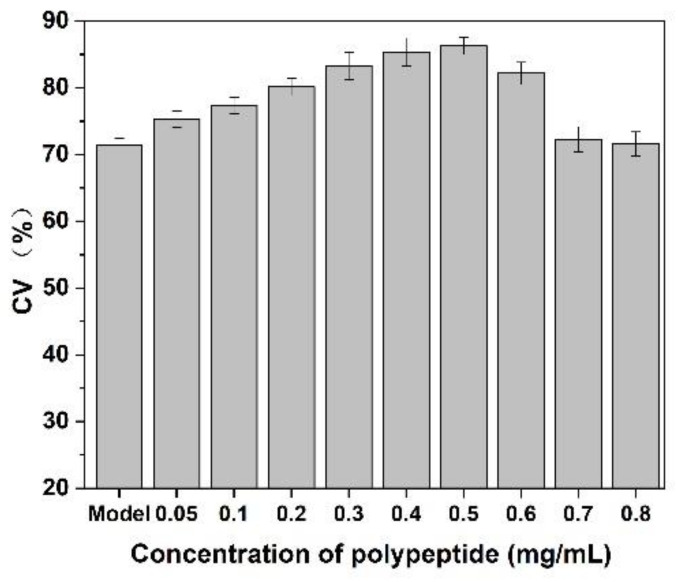
The effect of *Cornus officinalis* peptides on the oxidative damage CEF cells. Experimental conditions: phosphate buffer (pH 7.4), at 37 °C.

As shown in Figure 9, after adding of *Cornus officinalis* peptides, the activity of CEF cells was significantly increased, and the cell activity of CEF cell was 20% higher than the control group when the concentration of peptides was 0.4 mg/mL level. It is suggested that the prepared peptide had cell proliferation activity due to their antioxidant activity being able to scavenging the free radicals generated during cell growth. As presented in Figure 10, the cell activity of the oxidative damage model of CEF cells was restored dramatically after the addition of *Cornus officinalis* peptides. The highest cell viability of the oxidative damage model of CEF cells was achieved when the concentration of the added *Cornus officinalis* peptides was 0.5 mg/mL. However, when the concentration of peptides in the sample was at a continuous increased level, the cell viability of CEF cell gradually decreased, indicating that the excessive concentration of peptides was hindering the proliferation of CEF cells. These results suggest that *Cornus officinalis* peptides have the ability to promote cell proliferation and repair oxidative damage to cells.

## 4. Conclusions

In this study, the yield of peptides from *Cornus officinalis* by a composite enzymatic hydrolysis method was optimized using a RSM methodology engaged with BBD. The optimum conditions for preparation of peptides were determined based on response surface methodology. The optimal yield of peptides was 36.18 ± 0.26%, which is close to the predicted yield by the RSM model. The prepared peptides had a small relative molecular weight and exhibited superior free radical and antioxidant scavenging ability. Moreover, the *Cornus officinalis* peptides can effectively repair oxidative damage CEF cells and promote the proliferation of CEF cells. These experimental results in this study will be practical help to provide evidence for effectively prepared of peptides from *Cornus officinalis*, and find potential applications in the production of novel, high-value-added products.

## Figures and Tables

**Table 1:** Three independent variables with their corresponding levels.

Independent Variables	Levels		
	−1	0	1
A—pH value	5.0	6.50	8.0
B—Temperature (°C)	30.0	47.50	65.0
C—The amount of enzyme (%)	0.10	0.30	0.50

**Table 2:** Box–Behnken response surface design scheme and results.

Run	Coded Variable Levels			Yield (%)
	A	B	C	
1	6.50	47.50	0.30	35.28
2	6.50	47.50	0.30	34.54
3	6.50	47.50	0.30	36.05
4	9.02	47.50	0.30	29.35
5	5.0	65.0	0.10	31.15
6	6.50	47.50	0.30	34.88
7	3.98	47.50	0.30	27.87
8	6.50	76.93	0.30	29.57
9	8.0	65.0	0.10	31.07
10	8.0	30.0	0.50	28.96
11	6.50	47.50	0.64	31.75
12	8.0	30.0	0.10	32.75
13	8.0	65.0	0.50	29.75
14	6.50	47.50	0.30	35.15
15	5.0	65.0	0.50	32.18
16	6.50	47.50	0.30	35.89
17	5.0	30.0	0.50	27.54
18	5.0	30.0	0.10	27.88
19	6.50	18.07	0.30	27.15

**Table 3:** Sequential model summary of yield.

Response	Source	Sum of Squares	df	Mean Square	F-Value	Sequential *p*-Value	Lack of Fit	R^2^
Yield (%)	Mean	18,869.13	1	18,869.13				
	Linear	13.40	3	4.47	0.4295	0.7349	0.0003 **	0.1051
	2FI	15.73	3	5.24	0.4485	0.7229	0.0002 **	0.2421
	Quadratic	137.46	3	45.82	147.38	<0.0001 **	0.5627	0.9670

Significant at ** *p* < 0.01.

**Table 4:** One-way Analysis of variance (ANOVA) of model for the yield of *Cornus officinalis* peptides.

Source	Sum of Squares	df	Meansquare	*F*-Value	*p*-Value
Model	166.59	9	18.51	59.54	<0.0001 **
A	2.88	1	2.88	9.26	0.0140 *
B	9.01	1	9.01	28.97	0.0004 **
C	3.89	1	3.89	12.52	0.0063 **
AB	9.68	1	9.68	31.14	0.0003 **
AC	4.20	1	4.20	13.53	0.0051 **
BC	1.84	1	1.84	5.93	0.0377 *
A^2^	70.61	1	70.61	227.13	<0.0001 **
B^2^	76.22	1	76.22	245.16	<0.0001 **
C^2^	3.85	1	3.85	12.39	0.0065 **
Residual	2.80	9	0.3109		
Lack of Fit	1.11	4	0.2777	0.8227	0.5627
Pure Error	1.69	5	0.3375		
Cor Total	169.39	18	/		
C.V %	1.77				
R^2^	0.9835				
Adjusted R^2^	0.9670				

Significant at * *p* < 0.05. Significant at ** *p* < 0.01.

**Table 5:** The molecular weight of the prepared peptides.

MW (Da)	<1000	1000~3000	3000~5000	>5000
Ration (%)	72.57	12.84	10.21	4.38

## Data Availability

All the data generated by this research are included in the article.

## Sample Availability

Samples of the compounds are not available from the authors.
